# A primary multiple pleomorphic rhabdomyosarcoma of the heart in an adult dog

**DOI:** 10.1186/s12917-023-03701-5

**Published:** 2023-08-30

**Authors:** Olga Szaluś-Jordanow, Michał Czopowicz, Agata Moroz-Fik, Marcin Mickiewicz, Andrzej Łobaczewski, Sylwia Tarka, Łukasz Koperski, Rafał Sapierzyński

**Affiliations:** 1 Department of Small Animal Diseases with Clinic, Institute of Veterinary Medicine, Warsaw University of Life Sciences-SGGW, Nowoursynowska Str. 159c, Warsaw, 02-776 Poland; 2 Division of Veterinary Epidemiology and Economics, Institute of Veterinary Medicine, Warsaw University of Life Sciences-SGGW, Nowoursynowska Str. 159c, Warsaw, 02-776 Poland; 3 Veterinary Clinic Auxilium, Królewska Str. 64, Milanówek, 05-822 Poland; 4 Department of Forensic Medicine, Medical University of Warsaw, Oczki Str.1, Warsaw, 02-007 Poland; 5 Department of Pathology, Medical University of Warsaw, Pawińskiego Str. 7, Warsaw, 02-106 Poland; 6 Department of Pathology and Veterinary Diagnostic, Institute of Veterinary Medicine, Warsaw University of Life Sciences-SGGW, Nowoursynowska Str. 159, Warsaw, 02-776 Poland

**Keywords:** Rhabdomyosarcoma, Heart, Neoplasms, Dog, Echocardiography

## Abstract

**Background:**

Heart tumors are rare in dogs. They can be benign or malignant. Clinical signs depend primarily on the location of the tumor and its effect on blood flow.

**Case presentation:**

An eleven-year-old crossbreed male dog lethargic and anorectic for previous 3 days was presented to the veterinary clinic. The focused ultrasound assessment with sonograms in trauma (FAST) revealed multiple tumors in the heart which were then confirmed in echocardiographic examination performed by a veterinary cardiologist. Due to the poor general condition and grave prognosis, the dog was humanely euthanized. The autopsy revealed numerous intracardiac tumors in all four heart chambers. No proliferative changes were found in other organs either in thoracic or abdominal cavity. Immunohistochemical examination was performed using formalin-fixed, paraffin-embedded tissue from heart masses. The antibodies against myoglobin, desmin, smooth muscle actin, vimentin, CD34, S100, and pan-cytokeratin (AE1/AE3) were used. Microscopically, the tumor was composed of fascicles of spindle-shaped cells with pale eosinophilic cytoplasm with round, oval, and focally elongated nuclei and one or two prominent nucleoli. The tumor cells showed strong diffuse cytoplasmic immunopositivity for myoglobin and vimentin and focal staining for desmin. Immunostainings for smooth muscle actin-SMA, CD34, pan-cytokeratin, S-100 protein were negative. The immunohistochemical staining pattern confirmed rhabdomyosarcoma.

**Conclusions:**

This is the first description of the primary multiple heart rhabdomyosarcoma in a dog.

## Background

Heart tumors (HT) are uncommon in companion animals, accounting for roughly 0.2% of all neoplasms. Whether primary or metastatic HT occur more often is unclear both in dogs and cats [1, 2]. Hemangiosarcoma (HSA), chemodectoma, and lymphosarcoma are the most common HT types in dogs while lymphoma is most prevalent in cats [3].

Clinical signs and their severity depend on the type, location, and size of the tumor. As long as there is no effect on the blood flow, HT are most often diagnosed incidentally during a routine echocardiographic examination performed e.g., in search for metastases from the spleen HSA [4].

Rhabdomyosarcoma (RMS) is a soft tissue tumor derived from mesenchymal tissue with myogenic differentiation and associated with the embryogenesis of striated muscle. RMS has been described in many animal species, including dogs [5–9], cats [10], horses [11], cows [12], sheep [13] and goats [14] but in all it is considered as a very rare tumor.

It can originate from both skeletal and heart striated muscle, which does not mean that these are the most common locations of the tumor [5]. In dogs, most RMS are located in the urinary bladder. This disease usually occurs in dogs up to 18 months of age which indicates a juvenile predisposition [6–9]. Other locations of RMS, both primary and secondary are heart [15–17], brain [18], spinal cord [19], orbit [20, 21], ovary [5], oral cavity including tongue [22] and maxillary gingiva [6], esophagus [23], larynx [24], and forelimb [25]. RMS is very aggressive with distant metastases developing early in the course of disease. Therefore, the mainstay of treatment is the combination of radical surgery and postsurgical chemo- and/or radiotherapy. Despite intensive treatment, overall survival time is short both in human and animal patients [6].

RMS can be classified histologically as pleomorphic, embryonic or alveolar. Embryonic and follicular forms occur in young patients and are collectively referred to as juvenile RMS, while pleomorphic forms occur mainly in adults. Nodules are “grape-like” in their appearance [6, 26].

These tumors may be mistaken for undifferentiated sarcoma depending on the phenotype of the variant. Immunohistochemical staining or electron microscopy are essential for an accurate classification [26]. RMS is an extremely rare HT in dogs [16, 17]. The occurrence of this tumor in the heart has been described only in a few publications in dogs, and such a multiple heart RMS as presented below is an extremely uncommon diagnosis not only in veterinary but also in human medicine.

## Case presentation

**Fig. 1:**
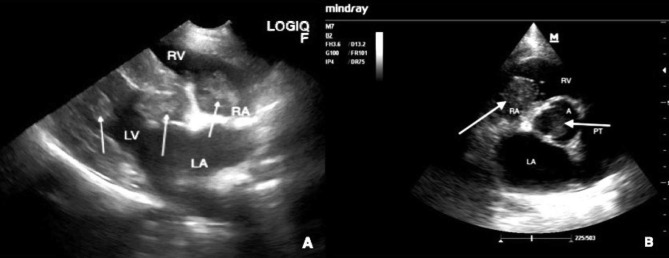
**A**. Focused echo, right parasternal long axis view. **B**. Echocardiography examination, right parasternal short axis view, arrows point into masses, A- aorta, PT- pulmonary trunk, LA- left atrium, LV- left ventricle RV- right ventricle, RA- right atrium

An eleven-year-old crossbreed male dog weighing 35 kg and living in a dog shelter was presented to the veterinary clinic semi-conscious in lateral recumbency. The dog had been lethargic and lacked appetite for previous 3 days. No other clinical sings were reported by the dog’s caretakers. In the clinical examination the rectal body temperature was 38.1 °C, mucous membranes were pink and moist, and lymph nodes were normal. Respiration and heart rate were increased to 45 breaths and 150 beats per minute, respectively. No pathological heart murmurs were audible. In the neurological examination reaction to external stimuli including pupils reaction to light was vastly reduced. The focused ultrasound assessment with sonograms in trauma (FAST) performed using GE Healthcare Logiq F6 (Chicago, USA) with a 10 − 6 MHz microconvex transducer revealed multiple masses in the heart and the patient was referred for a specialist echocardiographic examination. Echocardiographic examination was performed using Mindray M7 (Shenzhen, China) ultrasound machine with a 4-2s MHz phased array transducerin in a lateral recumbency. Numerous intracardiac tumors in all four chambers were found. The smallest nodule was approximately 0.5 cm in diameter and the largest was approximately 3.0 × 1.5 cm (Fig. 1). Two nodules in the left ventricle, one in aorta at the height of aortic valve and one in the right atrium were visualized. The echogenicity of tumors was uniform. The clinical diagnosis of a disseminated HT of atypical echocardiographic appearance was established. Due to poor general condition and grave prognosis the dog’s caretakers opted for euthanasia of the dog without any further diagnostic investigation.

The autopsy was performed immediately after euthanasia. No proliferative lesions were found in internal organs except for the heart either in the thoracic or abdominal cavity. Neither were the musculoskeletal masses detected in the anatomopathological examination. Detailed cardiac examination was performed as follows. First, the pericardial sac was gently removed. First incision was made along the long axis of the right atrium. Then, the right ventricle was cut along the interventricular septum and the left part of heart was examined starting form incision along the left atrium and incision made along the left ventricle at the interventricular septum.

**Fig. 2:**
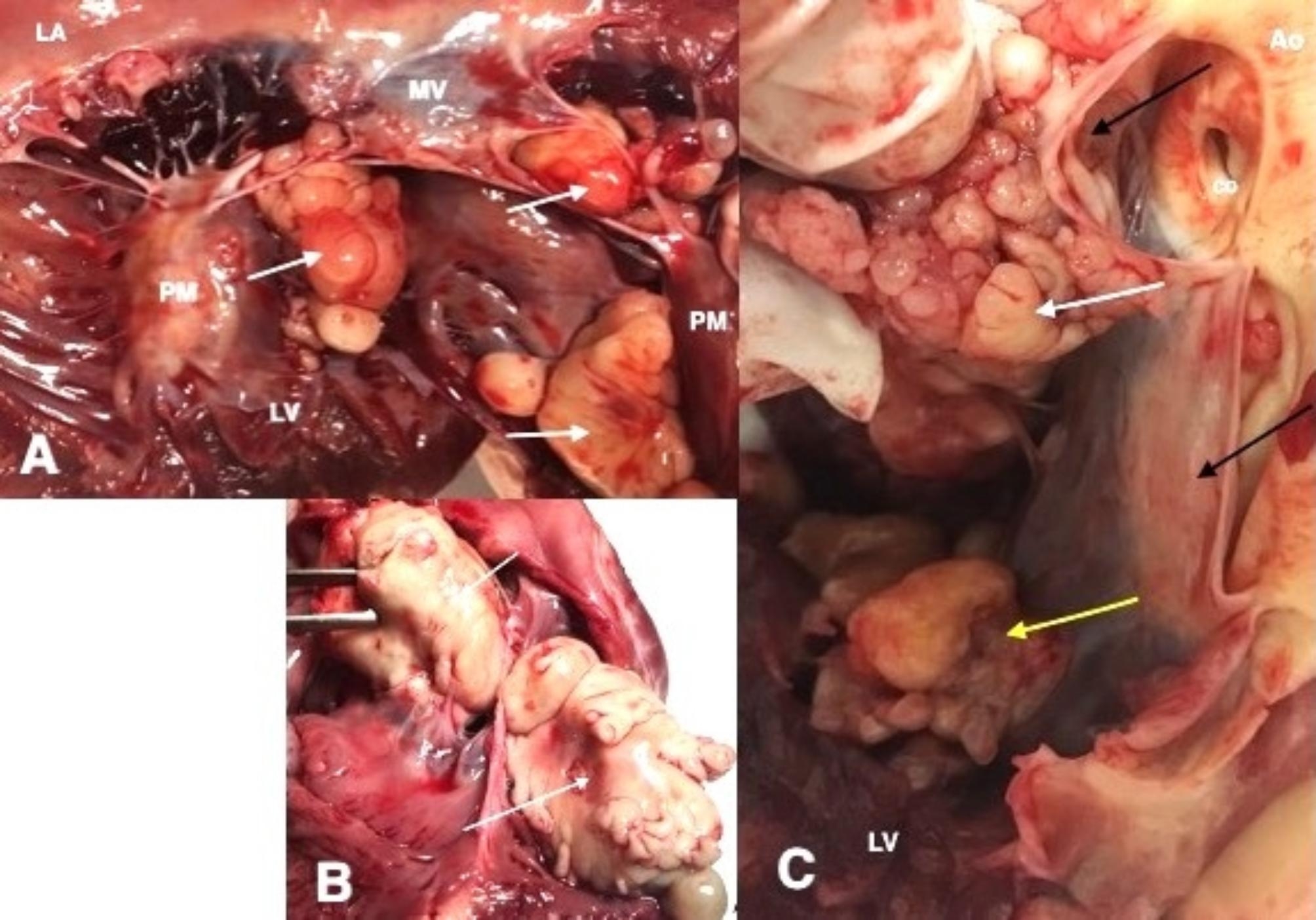
**A**. Multiple masses in left ventricle, arrows point into masses. **B**. Masses in right atrium. The left mass is attached to the tricuspid valve. **C**. Black arrows points into aortic valve, white arrow into mass attached to aortic valve, yellow arrow into mass attached into left ventricle. Ao- aorta, LA- left atrium, LV- left ventricle, PM- papillary muscles, MV- mitral valve, CO- coronary orifice

**Fig. 3:**
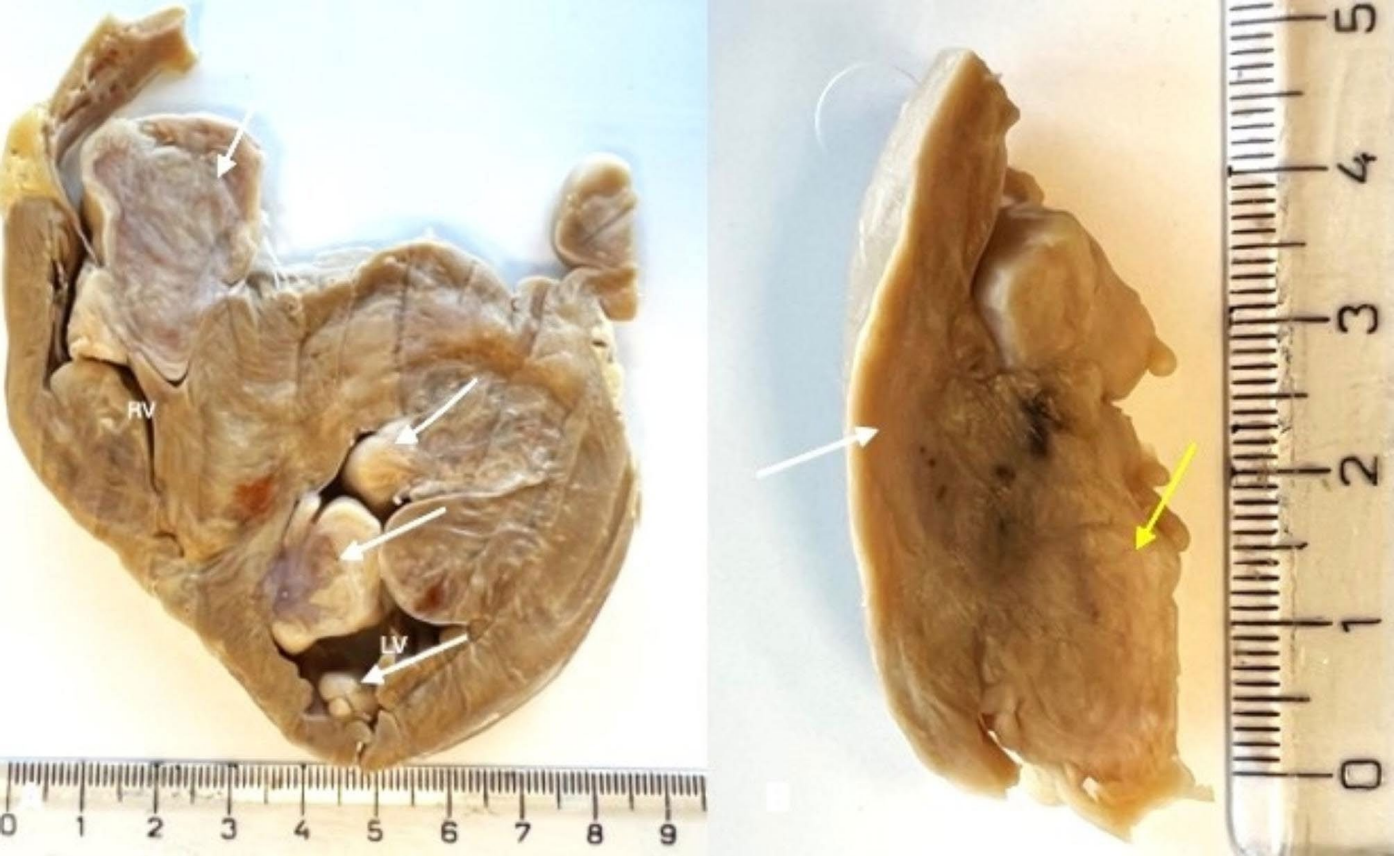
**A**. Cross section through the ventricles of the heart (formalin-fixed tissue). RV- right ventricle, LV- left ventricle. Arrows point into masses. **B**. Cross section through the right ventricular wall and tumor. The white arrow points to the wall of the right ventricle, the yellow one to the tumor. Visible measure in centimeters

Numerous nodular lesions found during dissection were located in all heart chambers. Nodules were fixed to the wall of the ventricles or atria or simultaneously attached to the valves and to the heart wall. The nodules varied from a few millimeters to a few centimeters in diameter and the largest was approximately 1.5 × 3 cm. In all cases, the base of the tumor was fixed to the walls of the heart chambers or to the valves (Figs. 2A-C and 3A-B). The masses were irregular in shape and yellowish in color.

**Fig. 4:**
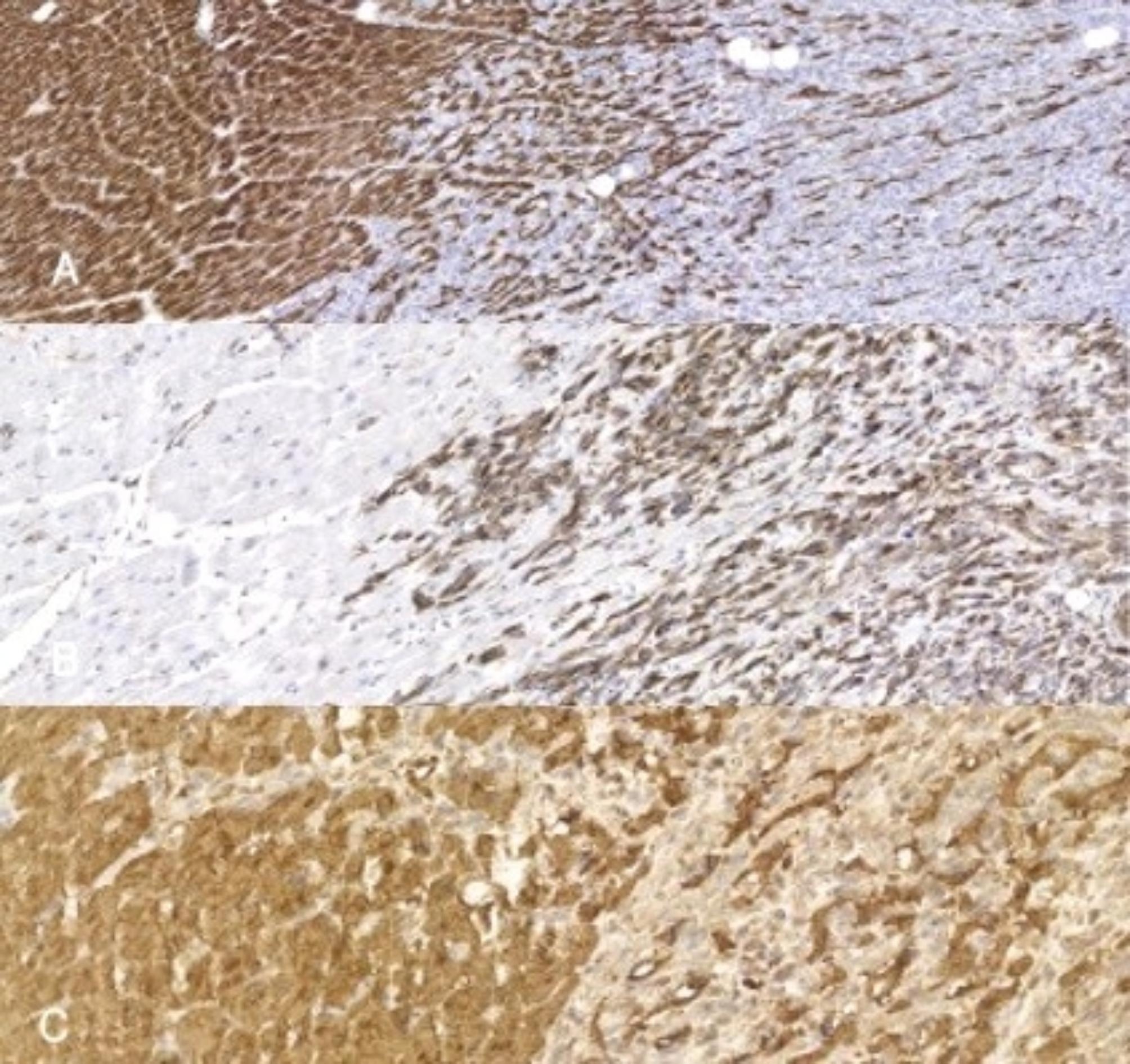
Cardiac rhabdomyosarcoma at the border with myocardial tissue; immunohistochemistry with antibodies: anti- desmin, anti-vimentin and antimyoglobin. Normal heart muscle is visible on the left, tumor tissue on the right. Visible infiltration of tissues one into the other. **A**. Desmin- multifocal strong cytoplasmic expression of desmin is visible in neoplastic cells and in normal cardiomyocytes. **B**. Vimentin- mild to strong cytoplasmic expression is visible in neoplastic cells and absent of expression in normal cardiomyocytes. **C**. Myoglobin mild to strong cytoplasmic expression is visible in neoplastic cells and in normal cardiomyocytes

Tissue samples were fixed in 10% buffered neutral formalin and embedded in paraffin, cut into 4 μm sections, and stained routinely with hematoxylin and eosin (H&E) as well as with a ready-to-use Masson’s Trichrome staining kit (Masson’s Trichrome Stain Kit; Polysciences, Inc., USA). On immunostaining with myoglobin, desmin, vimentin, the normal myocardium (Fig. 4 on the left) was visible adjacent to the tumor mass (Fig. 4, on the right). The tumor masses had no capsule or defined margin.

Immunohistochemical examinations were performed using formalin-fixed, paraffin-embedded (FFPE) 4 μm tissue sections and an automated immunostaining procedure (Dako Autostainer, Ft. Collins, USA). CD34, S-100, CD31 stains were performed on the BOND MAX immunohistochemistry apparatus (Leica Biosystems Deer Park, USA) in a closed system using the BOND Compact polymer antibody RTU detection system (ready to use). Masson’s Trichrome staining was performed on the Artisan apparatus (Agilent, Santa Clara,USA) a closed system, using Masson’s Trichrome reagents, catalog number AR173. SMA, VIM, Desmin and Multicytokeratin stains were performed on the Autostainer Link apparatus (Agilent, Santa Clara,USA) using the EnVision FLEX detection kit (Agilent, Santa Clara,USA). The antibodies from Agilent/Daco (Santa Clara, USA) against myoglobin (A 324), desmin (clone D33), smooth muscle actin (SMA) (clone 1 A4), vimentin (clone V9), CD34 (QBEnd 10) S100 (polyclonal), and cytokeratin (clone AE1/AE3). Myoglobin was used at a dilution of 1:30, the rest of antibodies were ready to use (RTU).

**Fig. 5:**
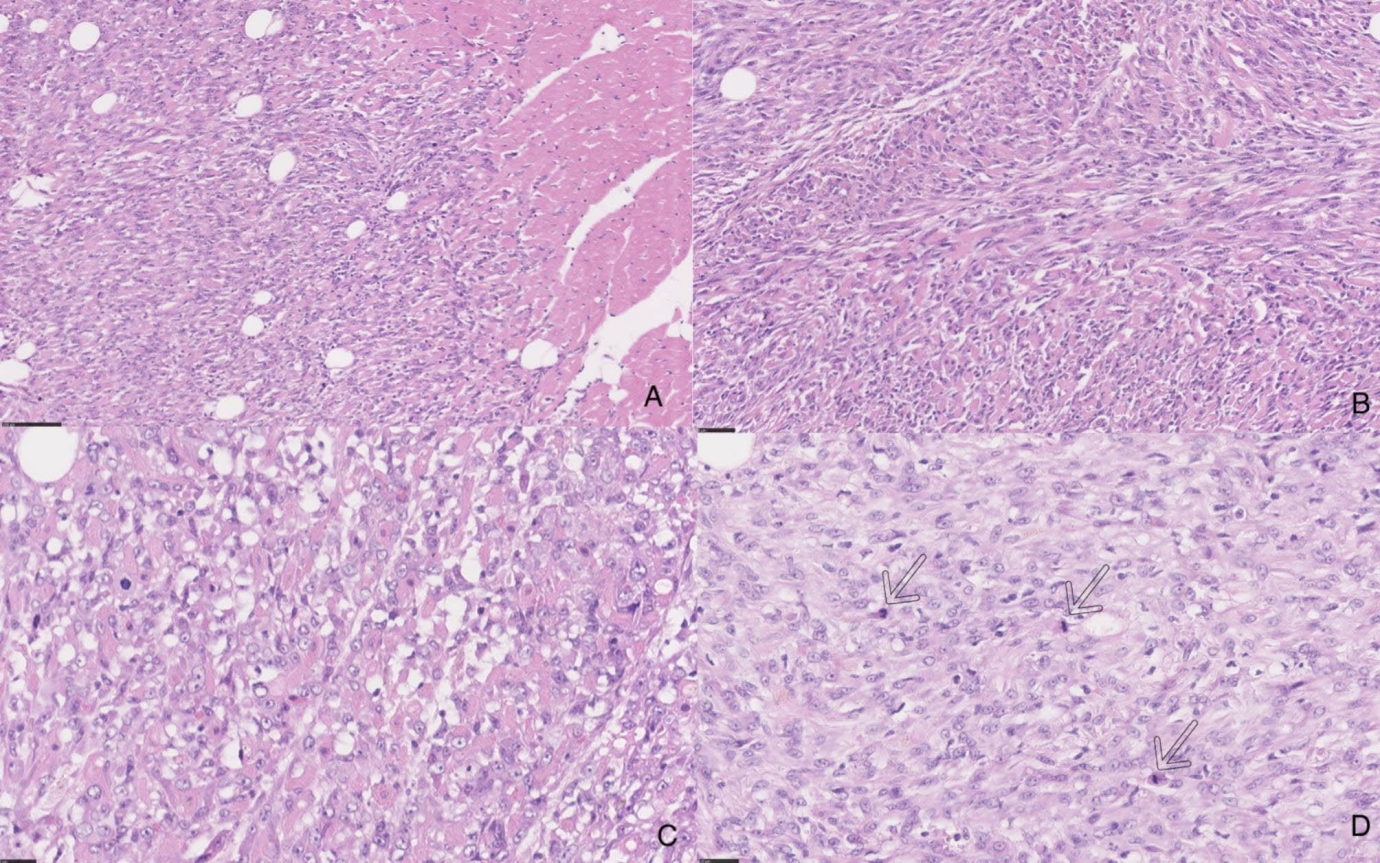
Cardiac rhabdomyosarcoma (H&E). (**A**) The spindle-shaped tumor cells; on the right normal cardiomyocytes, at low power. (**B**) The fascicles of tumor cells, at medium power. (**C**) Focus of more rounded and epithelioid tumor cells (**D**) Mitotic figure (arrows)

**Fig. 6:**
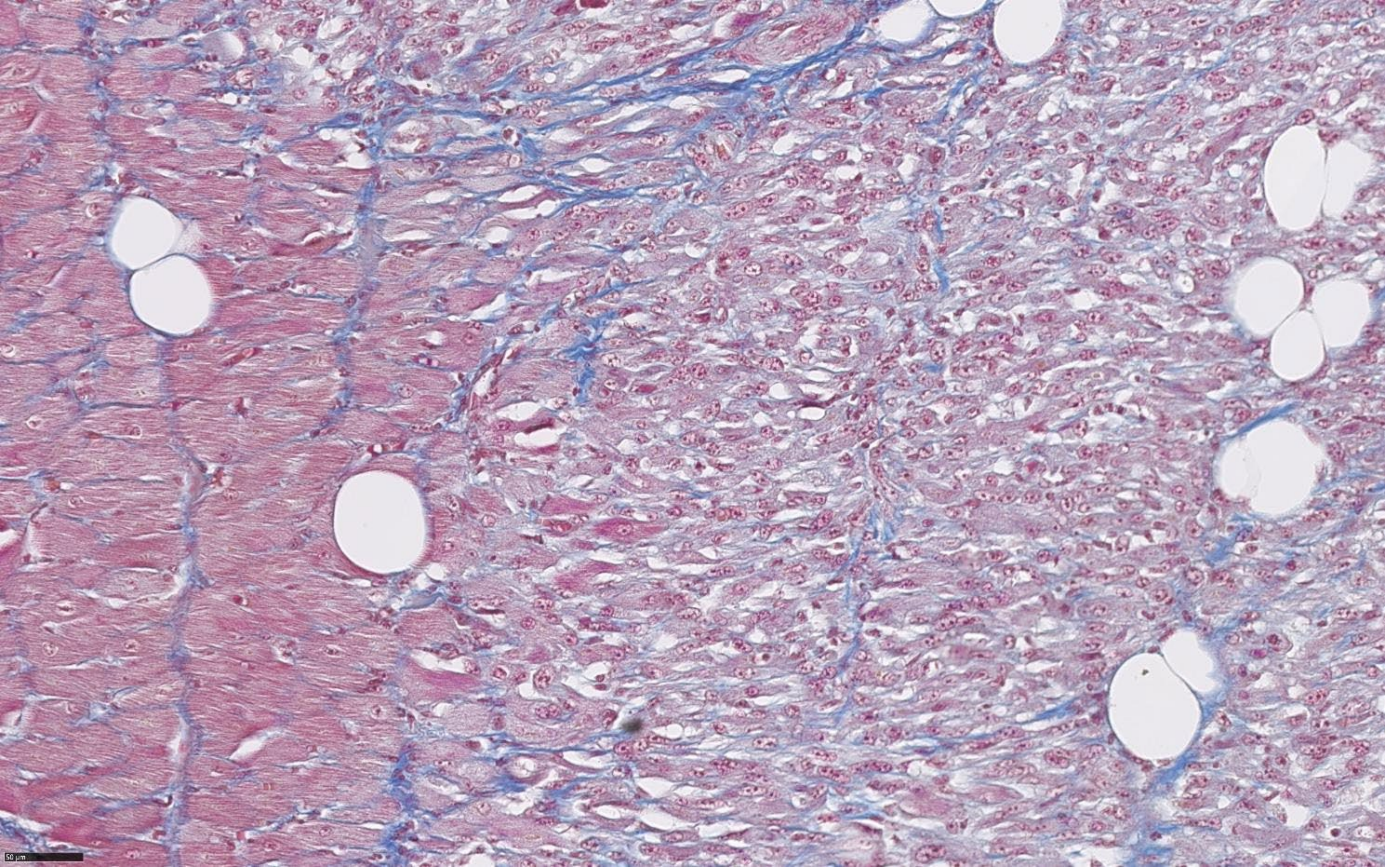
Cardiac rhabdomyosarcoma (Masson trichrome stain) – neoplastic cells are visible on the right, normal cardiomyocytes on the left

Microscopically, the tumor was composed of fascicles of spindle-shaped cells with pale eosinophilic cytoplasm with round, oval and focally elongated nuclei with anisokaryosis and one or two prominent nucleoli (Fig. 5A and B). In certain areas of the tumor, cells appeared more rounded and epithelioid (Fig. 5C) and the foci of tumor necrosis were observed. The number of mitotic figures was 2.6 per 2.37 mm^2^ (including atypical mitoses; Fig. 5D). Based on tumor differentiation, mitosis count and tumor necrosis, histologic grade 3 was established. The neoplastic cells stained as red or reddish using Masson’s trichrome staining method (Fig. 6).

The tumor cells showed strong diffuse cytoplasmic immunopositivity for myoglobin (Fig. 5A) and vimentin, and focal staining for desmin (Fig. 5B). Immunostaining for smooth muscle actin-SMA, CD34, pan-cytokeratin, and S-100 protein was negative.

Multiple cardiac pleomorphic RMS was diagnosed based on histopathological and immunohistochemical examination.

## Discussion and conclusions

In veterinary medicine heart masses are diagnosed using the echocardiographic examination, however, the definitive diagnosis of the type of tumor is only possible if postmortem examination has been performed. In addition, in the differential diagnosis, other types of heart masses, such as thrombi, vegetations or tuberculomas, should be taken into account [27, 28]. One of the most common cardiac sarcomas in dogs is angiosarcoma [3]. This tumor is most often located in the right atrium, however disseminated form or located in other structure, such as pericardial sac [29, 30] have also been described. Prognosis is poor and survival time vary from a few days to a few months [29, 30]. Similarly, in human medicine the most common primary sarcoma in the heart is angiosarcoma (37%), followed by malignant fibrous histiocytoma (24%), leiomyosarcoma (9%), rhabdomyosarcoma (7%), unclassified (7%) and others (16%) [31]. Primary malignant HT are rare both in human and veterinary medicine. As a result, even in human medicine large-scale epidemiological studies on their occurrence or randomized control trials and meta-analyses on their treatment are lacking [32]. Moreover, cytological assessment of sarcomas can be challenging. In up to 40% of people diagnosed initially with cardiac sarcoma, the initial diagnosis is modified in the referral oncological centers [33]. Surgical treatment of some types of neoplasms, such as myxoma in humans is possible, but due to high costs and poor prognosis, it is rarely performed in veterinary medicine [34]. In dogs with heart base tumors, the use of toceranib phosphate (Palladia®, Zoetis, USA) or radiation therapy has been shown to extend expected survival time, however the long-term prognosis for HT is generally poor to grave. People with cardiac sarcomas also have a poor prognosis. It depends on the type, size, location and, above all, on the influence on the hemodynamics of blood flow and the presence of metastases. Survival time in humans varies from a few days to two years. Most human cardiac RMS are single tumors located in the left atrium. Less often, can it be also detected in the right atrium or in the ventricles [35]. Multiple RMSs are very rare in humans. Some HT have malignant histopathological characteristics but do not metastasize and some are locally aggressive and infiltrative. This seems likely in our case. The high malignancy of the tumor was probably the reason for so many local metastases from the primary HT. Surgery combined with chemotherapy is the treatment of choice for this type of tumor, however the prognosis is poor and the median survival time is one year [35, 36]. There are no such reports in veterinary medicine. In a few publications describing the occurrence of canine cardiac RMS animals have been euthanized just after the initial diagnosis like in our case [16, 17]. This is the first description of the multiple pleomorphic cardiac RMS in a dog.

## Data Availability

Raw data supporting the findings of this study are available from the corresponding author [OSJ] on request.

## List of abbreviations

HSA: Hemangiosarcoma
HT: Heart tumors
RMS: Rhabdomyosarcoma
